# *Staphylococcus aureus* Infection Influences the Function of Intestinal Cells by Altering the Lipid Raft-Dependent Sorting of Sucrase–Isomaltase

**DOI:** 10.3389/fcell.2021.699970

**Published:** 2021-08-13

**Authors:** AhmedElmontaser Mergani, Dalanda Wanes, Natalie Schecker, Katja Branitzki-Heinemann, Hassan Y. Naim, Maren von Köckritz-Blickwede

**Affiliations:** ^1^Department of Biochemistry, University of Veterinary Medicine Hannover, Hanover, Germany; ^2^Research Center for Emerging Infections and Zoonoses (RIZ), University of Veterinary Medicine Hannover, Hanover, Germany

**Keywords:** *S. aureus*, human sucrase–isomaltase, brush border membranes, lipid rafts, sucrase–isomaltase

## Abstract

*Staphylococcus aureus* is an important nosocomial and community-acquired facultative intracellular pathogen. Many studies have reported that *S. aureus* infections are associated with intestinal symptoms, but little is known about the molecular mechanisms implicated in *S. aureus*-induced alterations of intestinal functions. In this study, we investigated the implication of lipid rafts in the interaction of *S. aureus* with Caco-2 cells. To assess potential alterations in the lipid raft structure and effects on the hydrolytic function, we utilized sucrase–isomaltase (SI) as the major intestinal α-glucosidase that is associated with and sorted to the apical membrane via lipid rafts. Seven days post-confluent, Caco-2 cells were infected with *S. aureus* Newman and further incubated for an additional 2 days. After 48 h, the levels of SI expression as well as the enzymatic function of this protein were assessed in the infected versus non-infected cells. Analysis of the sorting behavior of SI to the apical membrane constituted another crucial aspect in studying the effects of *S. aureus* on Caco-2 cells. For this purpose, the apical membranes or brush border membranes (BBMs; referred to as P2 fraction) were separated in both infected and non-infected cells from the basolateral and intracellular membranes (referred to as P1 fraction) by employing a cationic-based procedure using CaCl_2_. The data show that there is no significant change in the overall expression levels of SI in the infected versus non-infected cells as assessed by Western blotting analysis using monoclonal anti-SI antibodies. By contrast, a significant decrease in the localization as well as the specific hydrolytic activities of SI toward sucrose and isomaltose (Palatinose) was observed in the BBM (P2 fraction) in Caco-2 cells 48 h post-infection. Concomitantly, the specific SI activities increased in the basolateral membrane/intracellular fraction (P1). Noteworthy, the specific activity of SI in the BBM of infected cells was markedly reduced as compared with that of the non-infected counterparts. The data accumulated from this study strongly suggest that infections with *S. aureus* influence the final step in the lipid raft-associated trafficking of human SI and thereby may trigger secondary functional gastrointestinal disorders.

## Introduction

*Staphylococcus aureus* is a very important nosocomial and community-acquired pathogen. Its infection can involve many organs, and it can cause a broad spectrum of infections, including boils, abscess formation, wound infection, endocarditis, osteomyelitis, and sepsis or septic shock. Furthermore, it is a frequent pathogen in foreign body infections (Lowy, 1998). It is also often responsible for toxin-mediated diseases, such as toxic shock syndrome, scaled skin syndrome, and staphylococcal foodborne disease (Plata et al., 2009).

Furthermore, *S. aureus* may cause nosocomial antibiotic-associated diarrhea (Lane et al., 2018). In this regard, decreased stomach acidity can promote the establishment of colonization because of passing the gastric-acid barrier of *S. aureus* (Yoshida, 1999). An overgrowth of bacteria in the intestine can occur, leading to enteritis and/or diarrhea. *S. aureus* is implicated in inflammatory bowel disease (IBD) because gut-derived *S. aureus* antigens could induce inflammatory responses (Lu et al., 2003). Additionally, toxins of *S. aureus* are associated with food poisoning, and the alpha toxin of *S. aureus* can perturb the barrier function of intestinal cells *in vitro* by altering the junctional integrity (Kwak et al., 2012).

The small intestine is the principal organ that is implicated in the digestion of micromolecular nutrients, which is achieved by two families of enzymes, the peptidases and disaccharidases (Hauri et al., 1985; Lin et al., 2012). Of particular importance is the digestion of starch, glycogen, sucrose, maltose, and several other carbohydrates in the intestinal lumen that is achieved by the concerted action of a family of microvillar enzymes, the disaccharidases. The digestion of α-glycosidic linkages of carbohydrates commences by salivary and pancreatic α-amylases and is continued in the small intestine by two major mucosal α-glycosidases, sucrase–isomaltase (SI; EC 3.2.148 and 3.2.1.10) and maltase-glucoamylase (MGAM; EC 3.2.1.20 and 3.2.1.3) (Conklin et al., 1975). The digestive capacities of SI and MGAM cover almost the entire spectrum of carbohydrates that are linked via α-1,2, α-1,4, and α-1,6 linkages and comprise the majority of the typical diet in children and adults. Reduced expression levels or complete absence of intestinal disaccharidases at the cell surface of the enterocytes is associated with carbohydrate maldigestion and malabsorption, most notably described in several cases of genetically determined congenital SI deficiency (CSID) (Moolenaar et al., 1997; Sander et al., 2006; Alfalah et al., 2009; Treem, 2012), which is a primary deficiency of the enzyme SI. SI is the most abundant glycoprotein in the intestinal brush border membrane (BBM) and is exclusively expressed in the enterocytes. Due to its high abundance and its wide substrate specificity, human SI is responsible for about 60–80% of maltase activity in the intestinal lumen (Lin et al., 2012). SI is a heavily N-glycosylated and O-glycosylated protein that is trafficked with high fidelity to the apical membrane (Hauri et al., 1979; Naim et al., 1988a). The sorting of SI is mediated via its association with cholesterol- and sphingolipid-enriched membrane microdomains, known as lipid rafts (LRs) (Alfalah et al., 1999), in the *trans*-Golgi network for which proper O-glycosylation is essentially required (Wetzel et al., 2009).

In addition to primary genetic SI deficiencies, secondary deficiencies can be also induced and occur later in life and are mediated by environmental factors, including bacterial infections that negatively influence the intestinal physiology in general, for example, in IBD. In fact, *S. aureus* infections and toxins produced by *S. aureus* have been shown to be associated with intestinal malfunctioning (Lu et al., 2003; Kwak et al., 2012).

Little is known about the molecular mechanisms implicating *S. aureus* infection of the intestine and how this infection elicits secondary carbohydrate malabsorption. In this article, we have examined the molecular mechanisms that could lead to SI secondary deficiencies and demonstrate that LRs are directly affected by the *S. aureus* infection and that this triggers delayed trafficking and subsequent reduced function of SI at the apical membrane.

## Materials and Methods

### Cell Culture

Human epithelial colorectal adenocarcinoma, Caco-2, cell line (ATCC^®^ HTB-3^TM^7) was used in the study. Cells were maintained in high glucose (4.5 g/L) Dulbecco’s modified Eagle medium (DMEM; Sigma, Darmstadt, Germany), supplemented with 10% heat-inactivated fetal calf serum (FCS; Gibco BRL, Grand Island, NY, United States), and 50 U/ml of penicillin, and 50 μg/ml of streptomycin (Sigma, Darmstadt, Germany). Caco-2 cells were grown on polystyrene six-well plates (Sarstedt, Nümbrecht, Germany) for 7 days post-confluence to receive optimal SI expression (Supplementary Figure 1). Incubations were performed in a tissue culture incubator at 37°C, 5% CO_2_ in water-saturated air.

### Invasion of *Staphylococcus aureus* Newman Into Caco-2 Cells

The bacterial strain used in this study was the widely used laboratory strain *S. aureus* Newman (GenBank accession number AP009351.1), the respective green fluorescent protein (GFP)-expressing strain (Boero et al., 2021) and the methicillin-resistant *S. aureus* (MRSA) strain USA300 (Berends et al., 2010).

**FIGURE 1:**
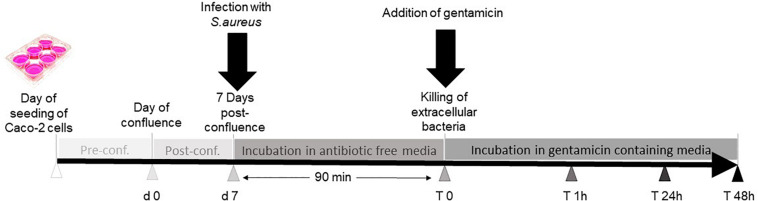
Work follow summary. Caco-2 cells were seeded in six-well plates. Seven days post-confluence, three wells were infected with *Staphylococcus aureus*, and the other three wells were not infected and served as controls. Thereafter, the cells were incubated in antibiotic-free medium for 90 min to allow the bacteria to enter the cells before replacing the medium with gentamicin-containing media to kill extracellular bacteria. The cells were incubated further for 48 h and then harvested for further analysis.

Gentamicin protection assay was used to determine intracellular bacteria after infection (see Figure 1). Briefly, bacteria were grown in brain heart infusion (BHI) medium at 37°C with shaking. On the day of infection, fresh overnight cultures were diluted 1:50 in BHI and then grown to mid-exponential growth phase (OD_600_ = 0.7). Ten milliliters of the bacterial culture was added to a 15-ml Falcon tube and was centrifuged at 3,800 × *g* for 10 min (Thermo Scientific Sorvall Legend X1R centrifuge, 4,500 rpm, Falcon rotor 75003658 Thermo Scientific; Thermo Fisher Scientific, Rockford, IL, United States) to discard the bacterial supernatant. The pellet was resuspended in 10 ml of antibiotic-free DMEM supplemented with 10% FCS. The Caco-2 cells were washed twice with antibiotic-free DMEM. Cells were infected with bacteria with multiplicity of infection (MOI) of 10, while negative control cells were left uninfected. After centrifugation of the six-well plates at 142 × *g* (1,000 rpm, plate rotor 75003624 M20; Thermo Fisher Scientific, Rockford, IL, United States) for 5 min, samples were incubated for 90 min at 37°C.

The total growth of bacteria was measured after 90-min incubation to determine the number of cells associated bacteria, using colony-forming unit (CFU) counting. Briefly, the infected Caco-2 cells and non-infected control cells were washed twice with DMEM with 10% FCS to remove non-associated bacteria followed by trypsinization for detachment of cells with 150 μl of trypsin-EDTA per well for 2 min. To each well, 350 μl of 0.1% Triton X-100 was added, and the Caco-2 cells were lysed by pipetting. Cell lysates were diluted and plated in duplicates on blood agar plates followed by an overnight incubation at 37°C. At the next day, colonies were counted, and percentage of associated bacteria at T0 (90 min of infection) was determined.

The cells were washed twice with 1 ml of antibiotic-free DMEM supplemented with 10% FCS to remove non-associated bacteria. For gentamicin treatment, DMEM supplemented with 10% FCS and 100 μg/μl of gentamicin was added to the cells. After further incubation for 1 h at 37°C, 25 μl of the supernatant of each well treated with gentamicin was plated on blood agar to ensure killing of bacteria by gentamicin. Furthermore, bacterial growth inside the cells was determined by counting CFU, as described above, 1, 24, and 48 h after gentamicin treatment. The percentage of invasive bacteria of compared with T0 (90 min) was determined.

### Cytotoxicity Assay

After 48 h of gentamicin treatment, lactate dehydrogenase (LDH) release assay was used to measure the viability of *S. aureus* infected and non-infected cells. Control cells were incubated with media only, whereas positive control cells were treated with 1% Triton X-100. Negative control was media without cells. Three microliters of the cell supernatants was added to 47 μl of LDH storage buffer [200 mM of Tris–HCl, pH 7.3, 10% glycerol, 1% bovine serum albumin (BSA)] and were mixed with 50 μl of detection reagent according to the manufacturer (LDH-Glo^TM^, Promega, Madison, WI, United States) and incubated for 60 min at 22°C in a 96-well plate. Luminescence was recorded using plate reader (Tecan, Grödig, Austria) as described in the manufacturer’s instructions.

### Live/Dead Cell Viability Staining of Caco-2 Cells After Infection With *Staphylococcus aureus*

Caco-2 cells were seeded on glass-bottom 24-well plates and were grown until 7 days post-confluence. The cells were infected as described above with *S. aureus* Newman GFP at the MOI of 10. Forty-eight hours after gentamicin treatment, infected cells and control cells were washed with 1 × PBS; 6.66 μl of ethidium homodimer-1 of the LIVE/DEAD^®^ Viability/Cytotoxicity Assay Kit was added to 3 ml of 1 × PBS. This solution was diluted 1:3 with 1 × PBS and was then added directly to the cells after addition of HOECHST in a dilution of 1:500. After an incubation of 10 min, the samples were analyzed by confocal laser scanning microscopy (CLSM) with the HCX μL APO apochromatic 40 × 1.25 glycerol immersion objective for correlation of infection with bacteria and cell death.

### Brush Border Membrane Preparation

Cellular homogenates of infected and non-infected Caco-2 cells were fractionated to apical membrane or BBM (P2 fraction), basolateral and intracellular membranes (P1 fraction), and the soluble and vesicular membranes (S fraction) using the divalent cation precipitation method (Schmitz et al., 1973; Naim et al., 1988b; Hein et al., 2011). The cells were homogenized in the hypertonic homogenization buffer (300 mM of mannitol and 12 mM of Tris–HCl, pH 7.1) supplemented with protease inhibitor mix (antipain 1.48 μM, pepstatin A 1.46 μM, leupeptin 10.51 μM, aprotinin 0.768 μM, soybean trypsin inhibitor 50 μg/μl, and phenylmethylsulfonyl fluoride (PMSF) 1 mM; all were obtained from Sigma, Darmstadt, Germany). The cellular homogenates were depleted from cell debris by centrifugation for 15 min at 5,000 × *g* (the supernatant referred to as H). This supernatant (referred to as H) was treated with 10 mM of CaCl_2_ at 4°C for 30 min with gentle agitation and centrifuged at 5,000 × *g* for 15 min to yield the pellet (P1). The supernatant was then centrifuged at 25,000 × *g* for 30 min to yield the pellet (P2) and the (S) fraction.

### Specific Enzyme Activity

Sucrase activity in H, P1, P2, and S fractions was measured using sucrose as a substrate (150 mM) essentially as described by Wanes et al. (2021); 25 μl of 150 mM of sucrose was added to 25 μl of sample and incubated for 1 h at 37°C followed by incubation for further 20 min at 37°C after adding 200 μl of GOD-POD monoreagent fluid (Axiom Diagnostics, Worms, Germany) to all samples, and the absorbance was measured at 492 nm. Sucrase specific activity was calculated by dividing the measured absorbance over the SI band intensity obtained from Western blotting of a similar sample volume.

### Detergent-Resistant Membranes or Lipid Raft Preparation

Lipid rafts or detergent-resistant membranes (DRMs) are known to play an important role in the activity and trafficking of SI. We therefore investigated the status of LRs upon infection of the Caco-2 cells by the *S. aureus* Newman. Forty-eight hours after gentamicin treatment, infected and non-infected control Caco-2 cells were solubilized with 1% (w/v) Triton X-100 in phosphate-buffered saline (pH 7.4) and protease inhibitor mix (mentioned above). The cellular lysates were centrifuged at 5,000 × *g* for 10 min at 4°C to remove cell debris. The supernatant was loaded on a discontinuous sucrose gradient and centrifuged as described by Wanes et al. (2021) to isolate the cholesterol- and sphingolipid-enriched LRs. Ultracentrifugation was performed at 100,000 × *g* for 18 h at 4°C using an SW 40 rotor (Beckman Coulter, Mississauga, ON, Canada). Fractions of 1 ml (10 fractions in total) were collected from the top of the gradient tube. LRs were recovered in the floating fractions of the gradient as assessed by the distribution of flotillin-2 (FLOT2), an LR scaffold protein marker, in Western blotting using anti-FLOT2 [FLOT2 (B-6, 1/6,000) from Santa Cruz Biotechnology, Inc., Dallas, TX, United States].

### Lipid Extraction and Lipid Composition Analysis

Total lipids were isolated from sucrose density gradients of Triton X-100 cellular extracts of bacterial infected or non-infected Caco-2 cells, as described previously (Brogden et al., 2014) based on the method of Bligh and Dyer (1959). Cholesterol analysis was performed with a Hitachi Chromaster HPLC, as previously described (Branitzki-Heinemann et al., 2016; Brogden et al., 2017).

### Western Blotting

Equal volumes of the fractions harvested from the sucrose-density gradients or 20 μg of H, P1, P2, and S of infected and non-infected cells were prepared for analysis by sodium dodecyl sulfate–polyacrylamide gel electrophoresis (SDS-PAGE) on 6 or 8% slab gels transferred onto a polyvinylidene difluoride (PVDF) membrane (Roth, Karlsruhe, Germany) essentially according to Wanes et al. (2021). FLOT2 was detected using purified mouse antibody [FLOT2 (B-6, 1/6,000) from Santa Cruz Biotechnology, Inc., Dallas, TX, United States]; and SI was detected using mAb anti-SI antibody HBB 3/705/60 (Hein et al., 2011). The secondary antibody was anti-mouse peroxidase conjugated secondary antibody (Thermo Fisher Scientific, Rockford, IL, United States). The protein bands were visualized via enhanced chemiluminescent peroxidase substrate and documented with a ChemiDoc MP^TM^ Touch Imaging System (Bio-Rad, Munich, Germany).

### Statistical Analysis

Experiments were carried out in duplicates or triplicates and repeated at least three independent times; GraphPad Prism version 8.0.1 (244; GraphPad Inc., San Diego, CA, United States) and Microsoft Excel 2016 (Microsoft, Redmond, WA, United States) software were used. Comparison of data was performed as described in the respective figure legends. *p*-values of 0.05 or less were considered as significant. Significant values with ^∗^*p* ≤ 0.05, ^∗∗^*p* ≤ 0.01, and ^∗∗∗^*p* ≤ 0.001.

## Results

### Non-cytotoxic Survival of *Staphylococcus aureus* Inside the Cells

In the present study, we evaluated the molecular modifications that occur in Caco-2 cells during *S. aureus* Newman infection. We first measured the percentage of invasive bacteria in three different time points (1, 24, and 48 h after gentamicin treatment), and we then tested the potential cytotoxicity due to infection.

**FIGURE 2:**
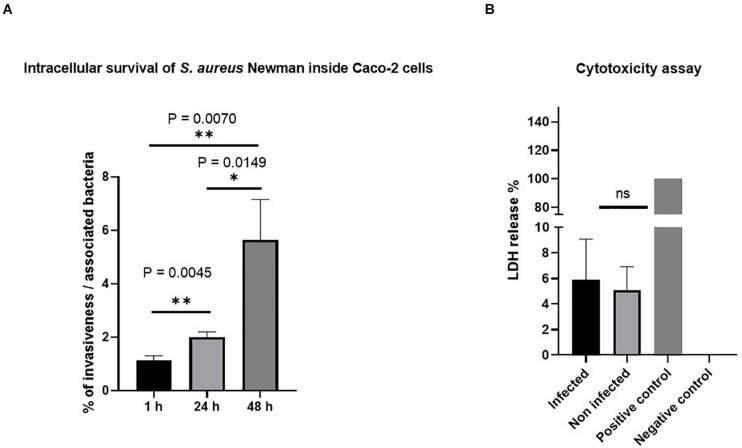
Invasion of *Staphylococcus aureus* Newman in Caco-2 cells. **(A)** *S. aureus* Newman-infected Caco-2 cells survived and multiplied inside the cells without affecting cell viability. The percentage of invasive bacteria showed significant differences between the three different time points after 1, 24, and 48 h of gentamicin treatment. The data were obtained from colony-forming unit (CFU) count of cell lysate infected with a multiplicity of infection (MOI) of 10. **(B)** The viability of Caco-2 cells was not affected upon *S. aureus* infection as estimated by lactate dehydrogenase (LDH) assay. Forty-eight hours post-infection, LDH release was measured in the supernatant of infected or non-infected Caco-2 cells or from cells lysed with 1% Triton X-100 that served as a positive control. ns, non-significant; ^∗^*p* ≤ 0.05 and ^∗∗^*p* ≤ 0.005 by using two-tailed Student’s *t*-test and using Tukey’s multiple comparison test. Data represent mean ± SEM of three independent experiments.

Figure 2 shows that *S. aureus* Newman successfully invaded the cells. Based on the lysate CFU count, the invasiveness percentage has been found to be about 1% at 1 h after gentamicin treatment, and increased to 2, and 5.5% in the next 24 and 48 h, respectively. We therefore conclude that the bacteria can invade and multiply within the cell.

To exclude the cytotoxic effect of the bacteria, we performed LDH assay and did not detect differences in LDH production 48 h after gentamicin treatment. Besides LDH, also viability assays using ethidium heterodimer for DNA staining of dead cells were used for microscopic analysis. We used these microscopy techniques since flow cytometry-based quantification by propidium iodide requires a detrimental detachment of cells. Representative images of this assay are shown Supplementary Figure 2. The images confirm that low percentage of dead cells occurs in non-infected control cells (A) as well as in infected cells (C), indicating that there is no specific cell death caused by invasive bacteria themselves.

Since the *S. aureus* USA300 strain showed cytotoxic effects after invasion of Caco-2 cells (Supplementary Figures 3, 4), the strain was not used for further experiments. To find effects of host–pathogen interaction on intestinal function independent of cell toxicity, it is the major idea to use a strain that does not result in significant cytotoxic effects. Therefore, all subsequent assays focused on *S. aureus* Newman strain.

### Effect of *Staphylococcus aureus* Infection on Sucrase–Isomaltase Sorting to the Apical Membrane

**FIGURE 3:**
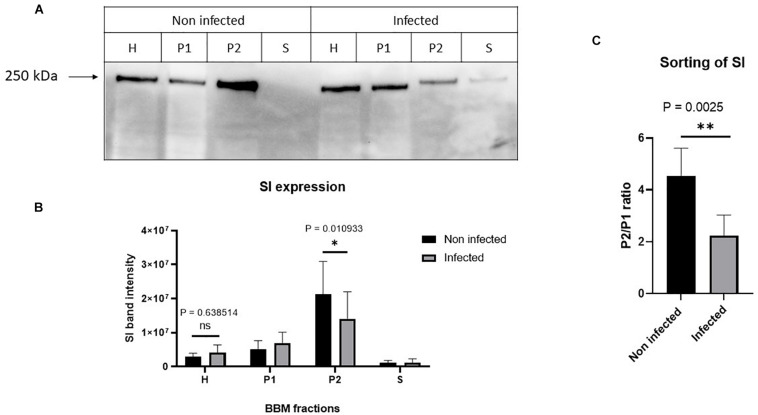
*Staphylococcus aureus* Newman infection of Caco-2 cells induced impaired trafficking of sucrase–isomaltase (SI) to the brush border membrane (BBM). Caco-2 cells were infected or not infected with *S. aureus* Newman. Forty-eight hours post-infection, Caco-2 cellular homogenates (H) were partitioned into intracellular and basolateral membranes (P1), brush border membranes (BBM) (P2), and vesicular and soluble fraction (S). Equal protein amount from the different fraction was analyzed by Western blotting using monoclonal anti-SI antibody **(A)**. While the infection did not significantly affect the overall expression of SI, the trafficking to the BBM (P2) was significantly reduced **(B)**. The sorting efficiency of SI to the BBM after infection with *S. aureus* was analyzed via comparing the expression of SI in the P2 versus P1. The ratio P2/P1 showed that the sorting of SI to the apical membrane is significantly reduced after *S. aureus* infection **(C)**. ns, non-significant; and ^∗^*p* ≤ 0.05 or ^∗∗^*p* ≤ 0.01 using two-tailed Student’s *t*-test. Data represent mean ± SEM of three independent experiments with samples in duplicates or triplicates.

**FIGURE 4:**
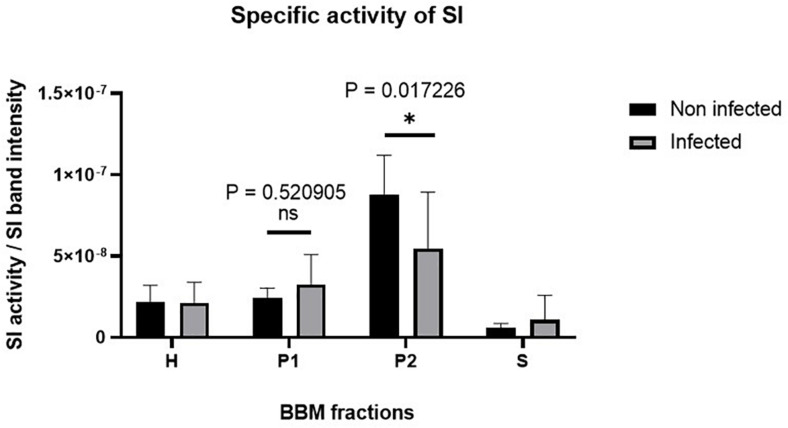
The specific activity of sucrase–isomaltase (SI) is substantially reduced in Caco-2 cells upon *Staphylococcus aureus* Newman infection. Caco-2 cells were infected or not infected with *S. aureus* Newman. Forty-eight hours post-infection, Caco-2 cellular homogenates (H) were partitioned into intracellular and basolateral membranes (P1), brush border membranes (P2), and vesicular and soluble fraction (S). Specific activity of SI in the different membrane fractions was analyzed and found to be significantly reduced in the P2 fraction in infected Caco-2 cells as compared with the non-infected. ns, non-significant; and ^∗^*p* ≤ 0.05 using one-tailed Student’s *t*-test. Data represent mean ± SEM of three independent experiments with samples in duplicates or triplicates.

Having excluded a potential cytotoxic effect of *S. aureus* infection on Caco-2 cells, the next question we addressed was whether the infected cells exhibit molecular and functional alterations. In a first set of experiments, we analyzed the function and expression of SI, a differentiation protein marker of intestinal Caco-2 cells, in the BBM. Here, we fractionated the cellular homogenates (H) of infected Caco-2 cells 48 h after treatment with gentamicin using CaCl_2_ (according to Schmitz et al., 1973, and the modification by Naim et al., 1988b) into BBM that are retained in the P2 fraction, intracellular and basolateral membranes (IM, in P1 fraction), and the cytosolic fraction (S). The expression of SI in these fractions was assessed by Western blotting using similar protein contents. The results shown in Figure 3 clearly indicate that the expression of SI in the BBM decreased significantly in the infected cells as compared with its counterpart in the non-treated cells. Obviously, this decrease is compatible with a substantial trafficking delay or altered sorting of SI to the basolateral membrane, since the infection itself did not impact the overall expression levels of SI as shown in the cellular homogenates. It rather resulted in an increase of SI in IM (P1) that is enriched in the endoplasmic reticulum (ER), Golgi, and basolateral membranes. In fact, the ratio of SI in BBM (P2) versus IM (P1) is reduced by almost two-fold upon infection, indicative of an intracellular retention or missorting of SI to the basolateral membrane (Figure 3C). Another critical parameter we examined is the effect elicited by the infection on the specific enzymatic activity of SI, which was significantly reduced in the P2 fraction (Figure 4).

### Lipid Raft Alterations Due to *Staphylococcus aureus* Infection

**FIGURE 5:**
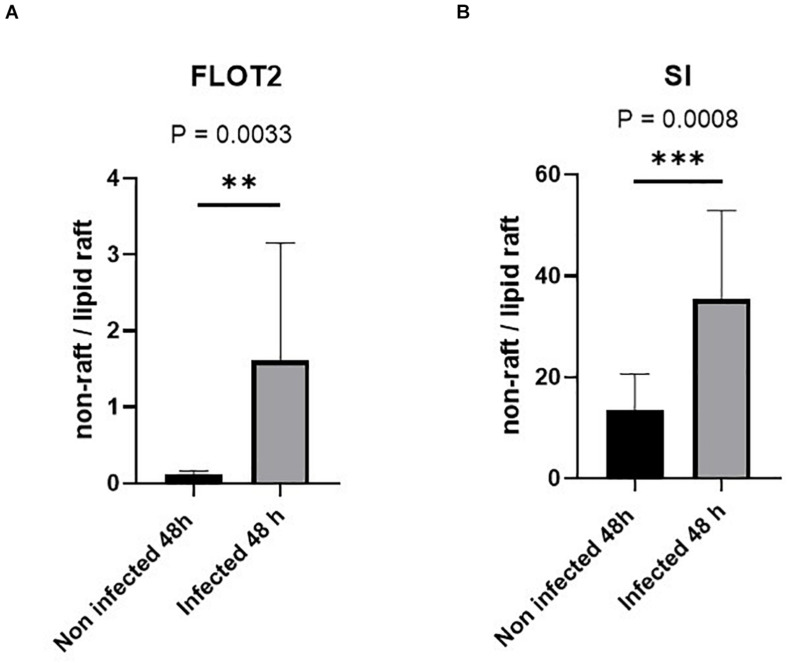
Lipid rafts (LRs) are altered in Caco-2 cells infected with *Staphylococcus aureus* Newman. Control non-infected or *S. aureus* infected Caco-2 cells were solubilized with 1% (w/v) Triton X-100 and separated on sucrose density gradients. Ten fractions were collected and used for further analysis in which the LRs are found in the top fractions (fractions 1–3) due to their low buoyant density. The distribution of the LR markers flotillin-2 (FLOT-2) **(A)** and sucrase–isomaltase (SI) **(B)** in the bottom three fractions (non-rafts) and in the top three fractions (LRs) in the control non-infected cells was compared with that of respective values from the same fractions in cells infected with *S. aureus* using by Western blotting. Raw data are shown in Supplementary Figures 5, 6. The redistribution suggests an altered LR composition in *S. aureus*-infected Caco-2 cells. Statistical significant differences are shown as ** *p* < 0.01 or ***p < 0.001.

Having demonstrated that the SI levels as well as its specific activity are reduced in BBM, we asked whether these effects are triggered by alterations in cholesterol- and sphingolipid-enriched membrane microdomains or LRs (Shimada et al., 2005; Lindner and Naim, 2009) in infected Caco-2 cells. We have previously shown that LRs are implicated in the apical sorting of SI in intestinal epithelial cells via O-linked glycans that act as a sorting signal (Alfalah et al., 1999). Furthermore, the functional capacity of SI is increased substantially when SI is associated with LRs (Wetzel et al., 2009). We therefore investigated the status of LRs upon *S. aureus* infection by examining the distribution of FLOT2, a LR scaffold protein marker (Langhorst et al., 2005; Browman et al., 2007), in sucrose density gradients of Triton X-100 detergent extracts of infected and non-infected Caco-2 cells. LRs are insoluble in Triton X-100 at 4°C, and by virtue of their buoyant density, they can partition into floating upper fractions of the gradient at low sucrose concentration. Expectedly, the majority of FLOT2 was retained in the top 3 fractions of the gradient, particularly in fraction 2, compatible with its association with LRs (Figure 5A and Supplementary Figure 5). A smaller proportion of FLOT2 was also found in the soluble non-LR fractions in the bottom of the gradient. Strikingly, upon infection, a substantial proportion of FLOT2 was redistributed to non-LR fractions 8 and 9 that resulted in a dramatic increase in the proportion of non-LRs versus LRs (Figure 5A and Supplementary Figure 5). An association of SI with LRs was also detected peaking in fraction 3 of the upper three fractions (Figure 5B and Supplementary Figure 6). Unlike FLOT2, the proportion of SI in the non-LRs was substantially higher than in the LRs. This observation reflects the biosynthesis and trafficking pathways of SI and its distribution in cellular membranes that are not enriched in LRs, such as the ER and several Golgi cisternae. Nevertheless, and similar to FLOT2, the proportion of SI in the non-LRs increased markedly upon infection (Figure 5). Together, these findings point to an alteration in the membrane lipid composition of LRs in infected cells.

**FIGURE 6:**
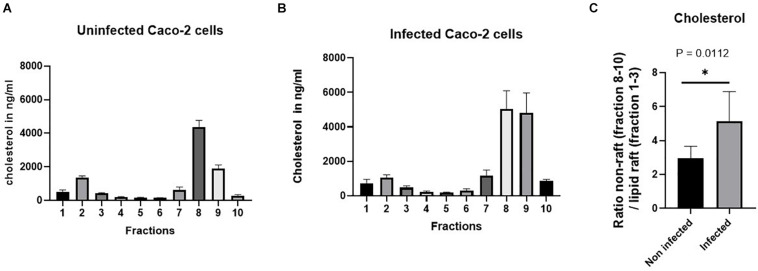
The proportion of cholesterol. For the separation of the lipid rafts (LRs) from the non-LR fractions, sucrose gradient fractionations were used, in which the LRs are found in the top fractions (fractions 1–3) due to their low buoyant density. The level of cholesterol in the bottom three fractions (non-rafts) and in the top three fractions (LRs) in the control non-infected cells was compared with that of cholesterol from the same fractions in cells infected with *S. aureus*. **(A,B)** The raw data of the proportion of cholesterol in the control non-infected cells compared with cells infected with *S. aureus*. Cholesterol analysis was performed by high-performance liquid chromatography (HPLC). **(C)** A significant reduction (almost 50%) in the cholesterol levels was observed in the LRs of Caco-2 cells infected with *S. aureus*. The data represent mean ± SEM of two independent experiments with samples in duplicates or triplicates (*n* = 5). Statistical significant differences are shown as *p < 0.05.

We therefore assessed the variations in cholesterol, one of the major two lipids in LRs, by comparing its content in the LRs versus non-LRs in non-infected and infected cells (see Figures 6A,B). Figure 6C shows that the ratio of non-LRs in the three bottom fractions of the gradient to the LRs in the upper three fractions is increased by almost two-fold in infected cells, compatible with a substantial reduction of cholesterol that normally assembles with sphingolipids to form LRs in cellular membranes. Since intact LR-enriched vesicles constitute an absolute requirement for efficient sorting of SI to the apical membrane (Alfalah et al., 1999), our data indicate that the reduced expression levels of SI in BBM are linked to delayed trafficking and missorting of SI to BBM due to distorted LRs (represented by P2 in Figure 3).

## Discussion

*Staphylococcus aureus* infections are among the most common human bacterial infections worldwide that affect several tissues, including the gastrointestinal tract. The current study sheds light on intestinal cell infection by *S. aureus* and the potential mechanism associated with the symptoms elicited by this infection. *S. aureus* Newman was chosen for experiments due to its origin from a human infection as well as its importance and frequency in laboratory use worldwide. This strain is methicillin-sensitive and displays robust virulence properties in animal models of disease and has already been extensively analyzed for its molecular traits of staphylococcal pathogenesis (Baba et al., 2008). Caco-2 cells were used as a suitable *in vitro* experimental model to study the inflammatory conditions caused by *S. aureus*. Previously, invasion and intracellular survival for 120 h of *S. aureus* in Caco-2 cells was observed for several *S. aureus* strains, including *S. aureus* RN6390 and *S. aureus* 502A. In these studies, the intracellular survival of *S. aureus* was associated with apoptosis (Hess et al., 2003) in contrast to our study, which demonstrated via CFU, LDH, and live/dead staining that *S. aureus* Newman strain can invade the intestinal cells and multiply inside the cells without causing significant cytotoxic effects at 48 h after infection. To find effects of host–pathogen interaction on intestinal function independent of cell toxicity, it was the major idea to use a time point and strain that does not result in significant cytotoxic effects. Accordingly, we hypothesized that this microorganism might induce alterations at the membrane and cellular levels that ultimately culminate in affecting the enzymatic function of intestinal cells leading to symptoms like those observed in carbohydrate malabsorption, such as diarrhea. The most prominent intestinal enzyme that is implicated in processing carbohydrates in the gut is SI. The digestive capacities of SI cover almost the entire spectrum of carbohydrates that are linked via α-1,2, α-1,4, and α-1,6 linkages and comprise most of the typical diet in children and adults. SI is synthesized and processed exclusively in intestinal cells. It is a heavily N- and O-glycosylated protein that is sorted almost exclusively to the apical membrane, where it exerts its digestive function. We show here that the high sorting fidelity of SI to the apical membrane is distorted in Caco-2 cells that have been infected with *S. aureus* as assessed by the substantial reduction of SI in the BBM fraction concomitant with an increase in the proportion of SI in the intracellular membrane and basolateral membranes. The switch of SI from the apical membrane to the intracellular and basolateral membranes suggests that the sorting machinery has been affected in the infected cells. The sorting mechanism of SI to the apical membrane occurs in the *trans*-Golgi network and utilizes cholesterol- and sphingolipid-enriched LRs (Alfalah et al., 1999). SI is packaged into specific LR-enriched sorting carriers that segregate from another vesicle type and trafficked along microtubules and the actin cytoskeleton to the apical membrane (Jacob and Naim, 2001; Jacob et al., 2003). As shown here, the infection with *S. aureus* has affected the LRs and has thus distorted the integrity of these vesicles, at least in part, impairing thus an efficient trafficking of SI to the apical membrane. The question that arises is whether the LR distortion and subsequently missorting of SI is a direct effect of *S. aureus* infection or is secondary to another mechanism that is affected by the infection. One potential mechanism could implicate actin depolymerization and redistribution as well as re-guidance of vesicle traffic as has been described for bacterial protein toxins (Schwan and Aktories, 2016). We have previously demonstrated that gliadin toxic peptides, which are implicated in the pathogenesis of celiac disease, interact directly with the actin cytoskeleton and elicit its depolymerization via competing in the binding of actin to Arp2/3 (Reinke, 2009; Reinke et al., 2011). The consequences are impaired trafficking of intestinal proteins that depend on an intact actin network. Structural alterations in the actin cytoskeleton that are associated with impaired trafficking to and reduced expression of SI at the cell surface have been also described in rotavirus-infected intestinal cells (Jourdan et al., 1998). Taking all this together, it can be postulated that an impaired trafficking of LR-enriched vesicles harboring SI—and other membrane proteins—and their intracellular retention leads to an intracellular accumulation of the two main lipid components comprising LRs, cholesterol, and sphingolipids. This in turn could elicit redistribution and alterations in the membrane lipid composition as has been demonstrated in several lysosomal storage diseases, such as Niemann-Pick Type C and Fabry diseases (Brogden et al., 2017, 2020; Shammas et al., 2019). In addition to altered membrane and protein trafficking in infected Caco-2 cells, *S. aureus* infection resulted in a reduction of the overall enzymatic activity of SI, most likely due to altered LRs. It has been shown before that association of SI into LRs increases activities of both sucrase and isomaltase, by almost three-fold, since SI clusters into LRs, exhibits a cooperative hydrolytic activity, and increases substrate accessibility (Wetzel et al., 2009). Reduced enzymatic activities of SI and also another LR-associated disaccharidase, maltase-glucoamylase, have been reported in intestinal biopsy specimens of patient with Niemann-Pick Type C, in which the trafficking or SI and LRs is markedly affected. The substantial reductions in the catalytic activity and cell surface expression of SI lead collectively to a substantial loss of function of SI at the cell surface. *In vivo*, these functional deficits can trigger symptoms, such as diarrhea, flatulence, and abdominal cramps, which are associated with functional gastrointestinal disorders (FGIDs), such CSID and irritable bowel syndrome. Unlike the secondary effects on SI function in *S. aureus* infection, FGIDs are genetically determined disorders in which SI is directly affected and constitutes therefore the primary cause of these diseases. The common features of these primary and secondary disease phenotypes are the impaired trafficking of SI and also altered association with LRs, which altogether lead to enzyme malfunction.

## Data Availability Statement

The original contributions presented in the study are included in the article/Supplementary Material, further inquiries can be directed to the corresponding author/s.

## Author Contributions

AM, DW, NS, and KB-H performed the experiments and interpreted the results. AM analyzed the data statistically and drafted the first version of the manuscript. HN and MK-B developed the concept of the study and analyzed the results. HN, MK-B, and DW wrote the final version of the manuscript. All authors contributed to the article and approved the submitted version.

## Conflict of Interest

The authors declare that the research was conducted in the absence of any commercial or financial relationships that could be construed as a potential conflict of interest.

## Publisher’s Note

All claims expressed in this article are solely those of the authors and do not necessarily represent those of their affiliated organizations, or those of the publisher, the editors and the reviewers. Any product that may be evaluated in this article, or claim that may be made by its manufacturer, is not guaranteed or endorsed by the publisher.
