# Supplementary material for: Staphylococcus aureus Infection Influences the Function of Intestinal Cells by Altering the Lipid Raft-Dependent Sorting of Sucrase–Isomaltase
Source: Front Cell Dev Biol. 2021 Aug 13;9:699970. doi: 10.3389/fcell.2021.699970 (PMC8418112; doi:10.3389/fcell.2021.699970)
Supplement: Supplementary file 1 [file Data_Sheet_1.PDF]

Supplemental Figures

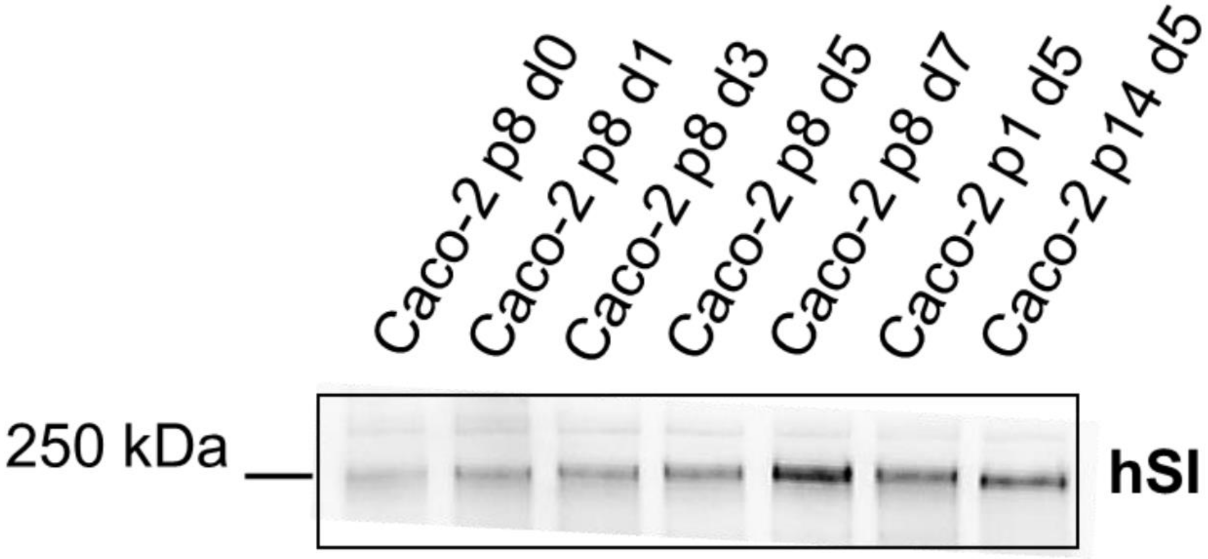

Supplemental Figure 1: Expression of human sucrase-isomaltase (hSI) at different days post-confluency. An important step in the investigation of the function of Caco-2 cells is the analysis of hSI expression. Caco-2 cells were lysed at day 0, day 1, day 3, day 5 and day 7 post-confluency. 50 µg protein were loaded on SDS-gels with 6% polyacrylamide. Shown are different states post-confluency: day 0, day 1, day 3, day 5 and day 7. The experiment was performed three times, shown is one representative Western blot. Day 7 was chosen for further experiments because of most abundant hSI expression at respective time post-confluency. The expression of hSI was verified for later passaging numbers (p), as for example for p14. Expression was also examined in the p1. This sample was used as a positive control.

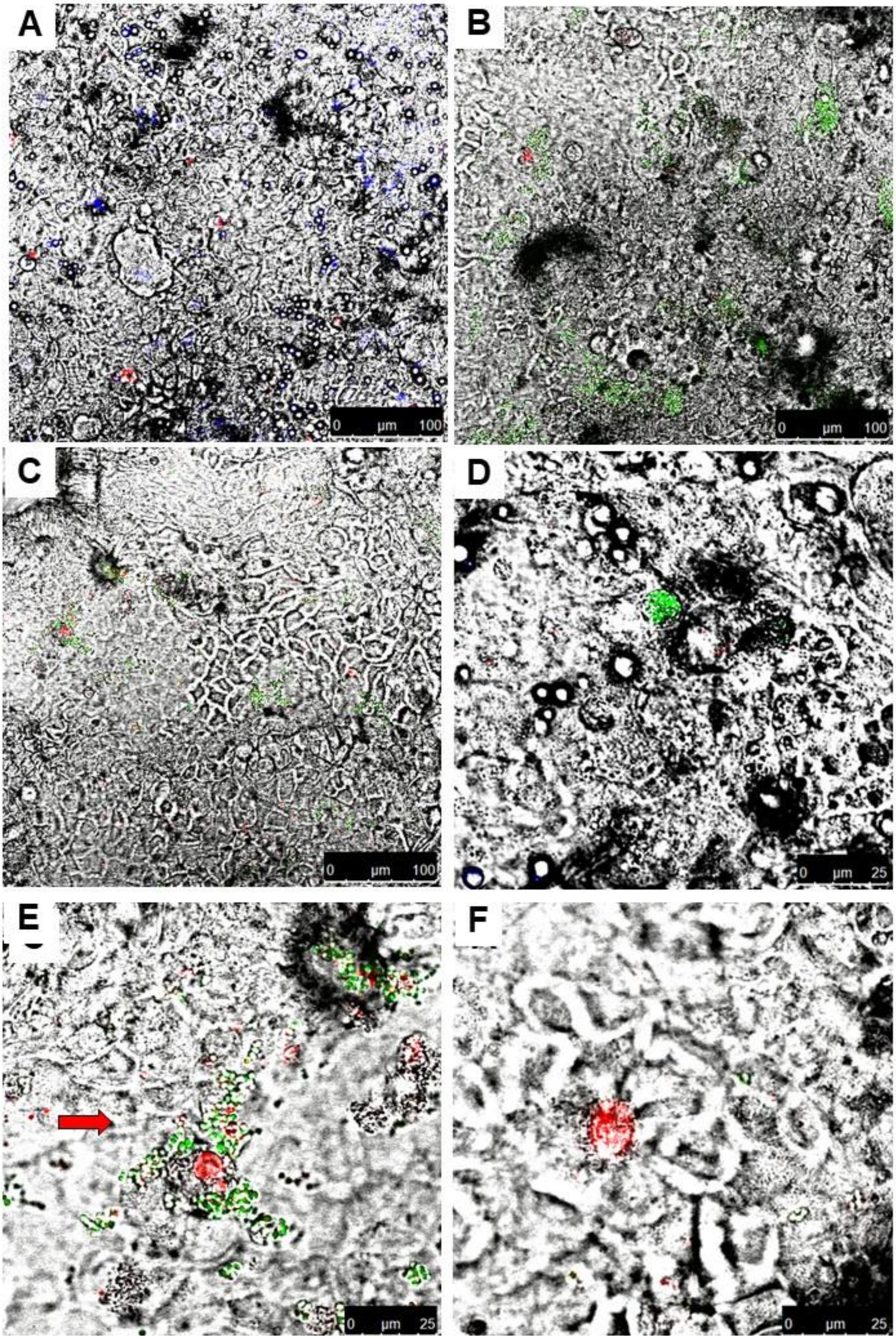

Supplemental Figure 2: Viability of Caco-2-cells after infection with S. aureus. (A) The micrograph of non-infected Caco-2 cells and **(B-F)** infected with GFP labeled *S. aureus* Newman at MOI 10 for 90 min. Hoechst staining of nuclei is shown in blue and *S. aureus* Newman GFP in green. Dead cells are stained with ethidium homodimer in red. The images show that low percentage of dead cells occurs in non-infected control cells (A) as well as in infected cells (C), indicating that there is no specific cell death caused by invasive bacteria themselves. Figure B-D show infected cell without cell death.

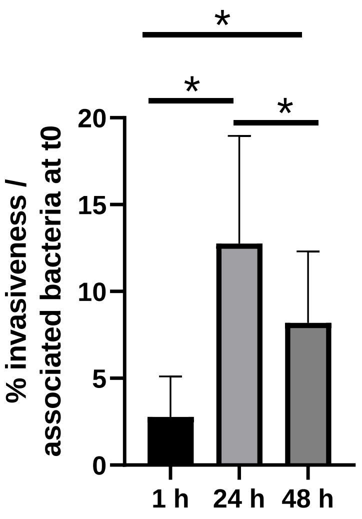

Supplemental Figure 3: Invasion of *S. aureus* MRSA USA 300 in Caco-2 cells. The data were obtained from CFU count of cell lysate infected with a MOI of 10 and show intracellular survival of *S. aureus* MRSA strain USA 300.

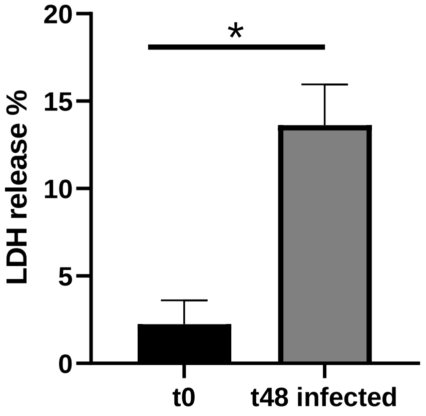

Supplemental Figure 4: The viability of Caco-2 cells was significantly affected upon MRSA infection as estimated by lactate dehydrogenase (LDH) assay. Forty-Eight hours post-infection, LDH release was measured in the supernatant of infected or non-infected Caco-2 cells. Cells lysed with 1% Triton X-100 served as a positive control (100%). Data show that infected Caco-2 cells exhibit significantly increased LDH release which is associated with cytotoxic effects and cell death.

N=1 1st fraction 10th fraction

Non-infected

Infected

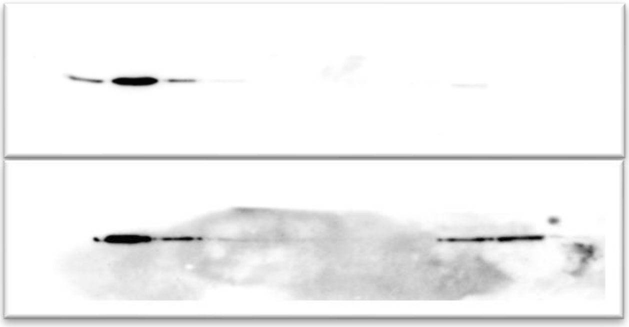

N=2:

Non-infected

Infected

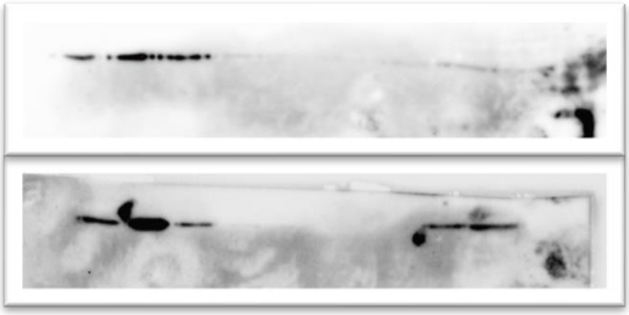

N=3

Non-infected

Infected

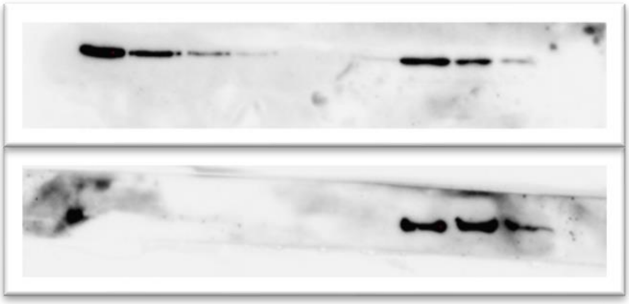

N=4

Non-infected

Infected

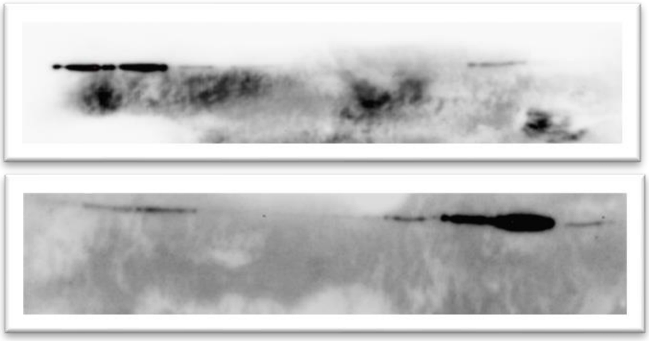

Supplemental Figure 5: Raw data of FLOT2-distribution in lipid rafts comparing control cells versus *S. aureus* Newman infected cells. Control non-infected or *S. aureus* infected Caco-2 cells were solubilized with 1% (w/v) TX-100 and separated on sucrose density gradients. In these experiments, the gradient has been fractionated into 10 equal 1ml fractions and equal volume of each fraction has been analyzed by SDS-PAGE and Western blot. The enrichment of FLOT2 in the fractions of the gradient is indicative for intact or possibly distorted lipid rafts and is used as reference protein to assess the distribution of sucrase-isomaltase (SI) shown in Supplemental Figure 2. As expected, FLOT2 is found in the floating LRs fractions 1–3 in the control

cells and very little in the soluble fractions 8. However, this pattern has changed in infected cells and FLOT2 was partially redistributed to fractions 2 and 3 in the floating fractions and to the soluble fractions 8 and 9. The ratio of non-LRs in the three bottom fractions of the gradient to the LRs in the upper three fractions of FLOT2 is increased upon infection as quantified and shown in Figure 5. The redistribution suggests an altered lipid rafts composition in *S. aureus* infected Caco-2 cells.

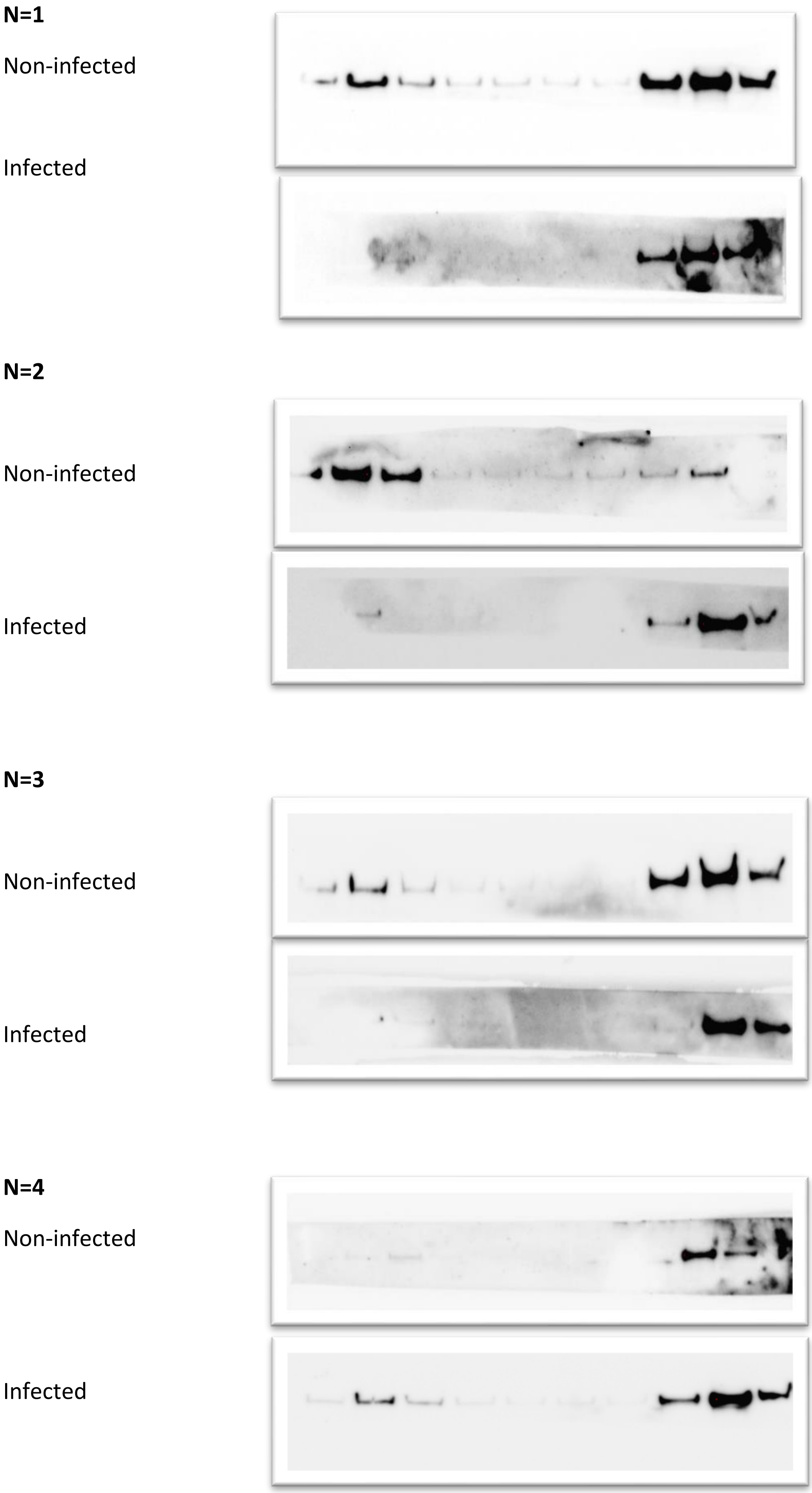

Supplemental Figure 6: Raw data of SI-distribution in lipid rafts (LRs) comparing control cells versus *S. aureus* Newman infected cells. Control non-infected or *S. aureus* infected Caco-2 cells were solubilized with 1% (w/v) TX-100 and separated on sucrose density gradients. Ten fractions were collected and analyzed for distribution of the lipid rafts marker SI by Western blotting. SI was found in the floating LRs fraction (1 -3) in

the control cells and to a greater extent in the soluble fractions 8-10. In the infected cells, however, SI was no more retained in the LRs fractions. The ratio of non-LRs in the three bottom fractions of the gradient to the LRs in the upper three fractions of SI is increased upon infection as quantified and shown in Figure 5. The redistribution suggests an altered lipid rafts composition in *S. aureus* infected Caco-2 cells.
